# Supplementary material for: Flagellin-Fused Protein Targeting M2e and HA2 Induces Innate and T-Cell Responses in Mice of Different Genetic Lines
Source: Vaccines (Basel). 2022 Dec 8;10(12):2098. doi: 10.3390/vaccines10122098 (PMC9786633; doi:10.3390/vaccines10122098)
Supplement: Supplementary file 1 [file vaccines-10-02098-s001.zip › vaccines-2007828-supplementary.pdf]

Supplementary Materials

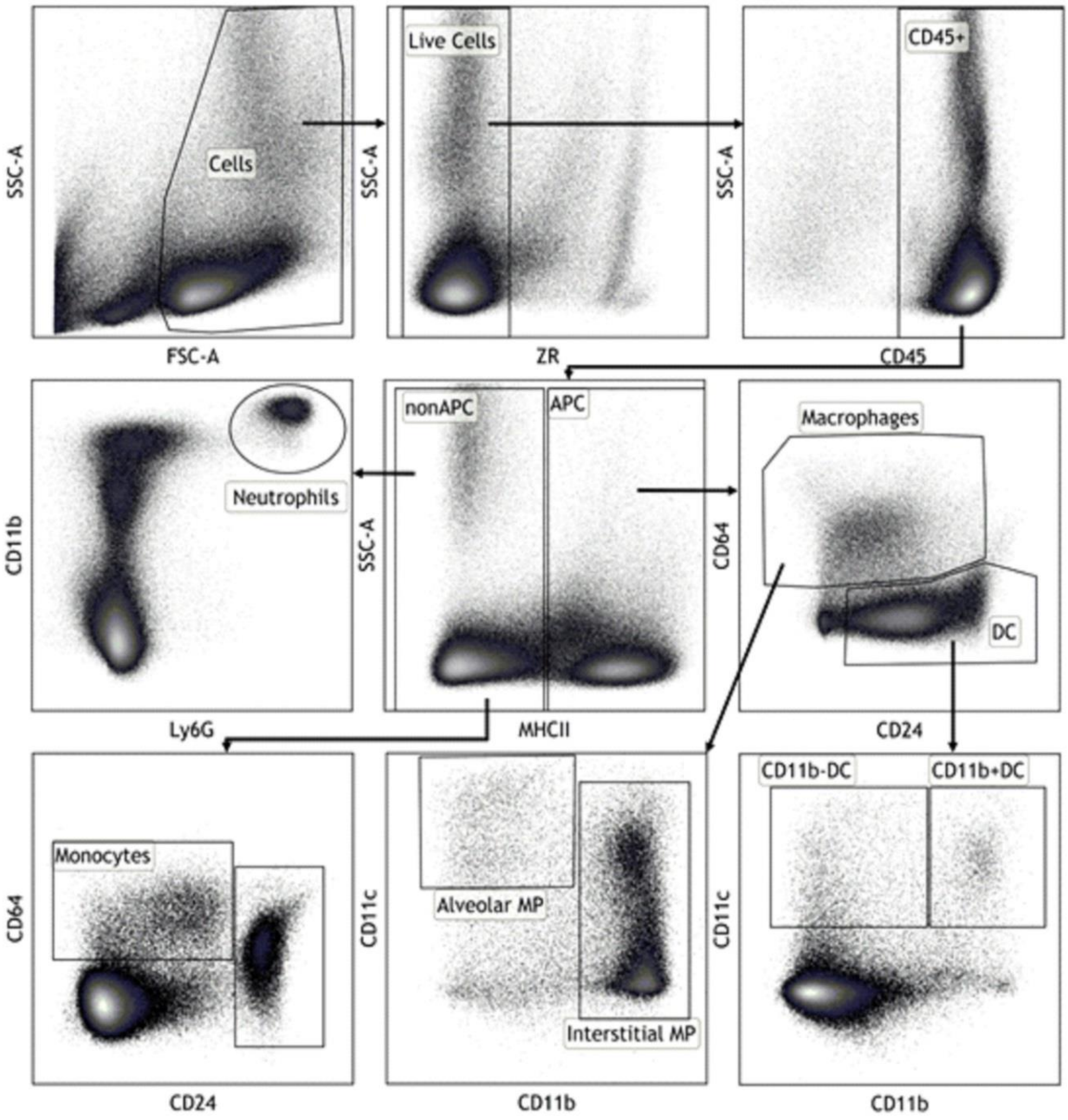

Figure S1. Tactics of gating cells of innate immunity. After pruning of FSC-A/FSC-H doublets (not shown) and isolation of the live single cell population based on light scattering characteristics (FSC-A/SSC-A -Cells) and Zombie Red dye binding (Live cells), immunocytes were gated by the presence of the CD45 marker (CD45+). Upon further analysis, the following cell populations were isolated: neutrophils - SSChiCD45+Ly6G+; monocytes -MHCII-CD64+CD24+; alveolar macrophages - MHCII+CD64+ CD11c+CD11b-; interstitial macrophages - MHCII+CD64+CD11b+ CD11c+/-; DC1-CD45+CD11c+CD11b-MHCII+CD64-CD24+ (CD11b-DC) and DC2 - CD45+CD11c+CD11b+MHCII+CD64-CD24+ (CD11b+DC).

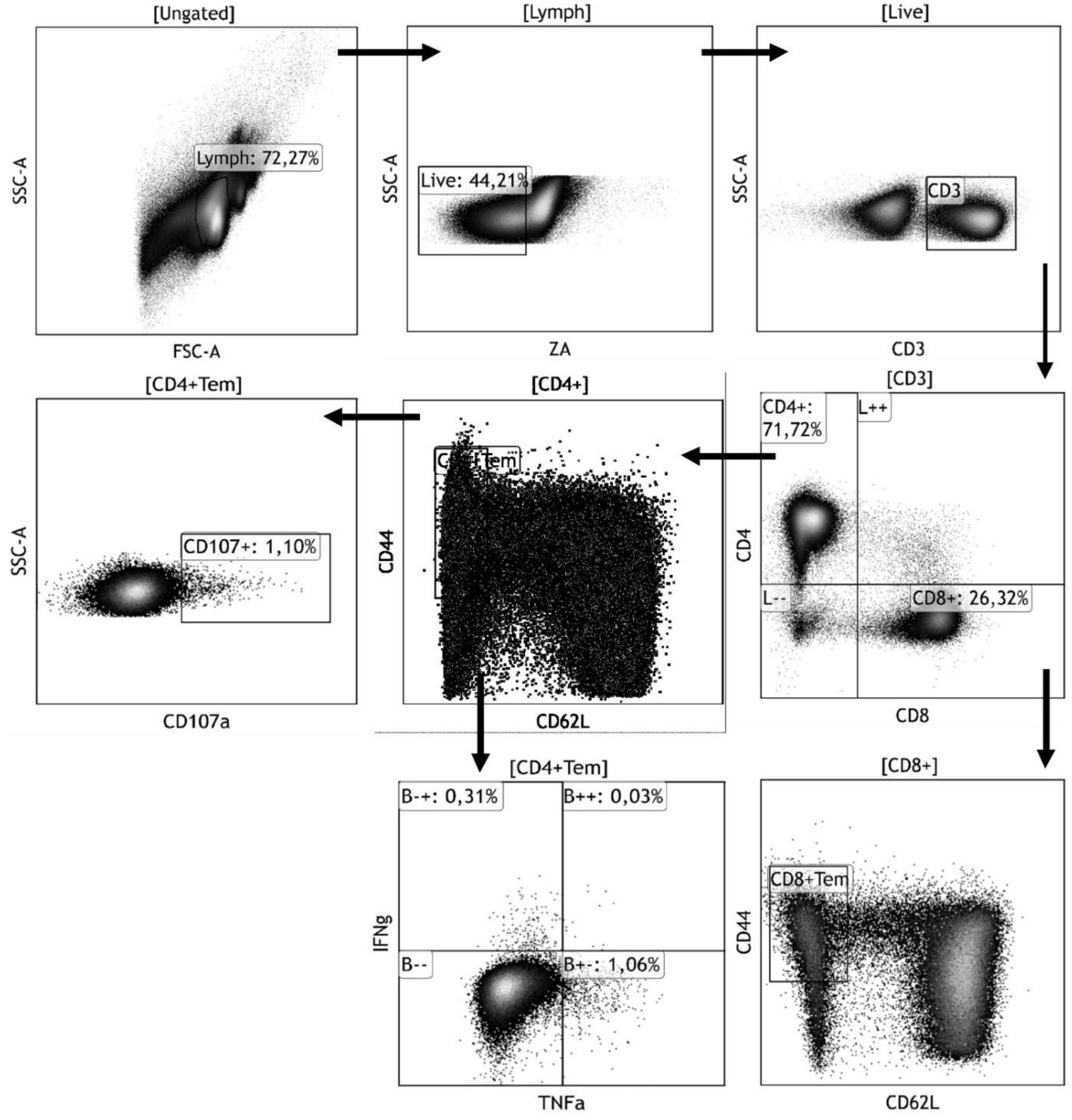

Figure S2. Sequential gating strategy for the evaluation of antigen-specific polyfunctional memory T cells in mouse lung homogenates. A population of lymphocytes (LY gate) was isolated based morphological features (size, granularity) in the FSC and SSC coordinates. Non-viable cells (Zombie Aqua positive events) were excluded from the analysis, and the population of live lymphocytes (Live gate) was divided into two main subpopulations of T-lymphocytes based on the constructed CD3+/CD4+ or CD3+/CD8+ gates: T-helpers (gate CD4+) and cytotoxic lymphocytes (gate CD8+). Further analysis of each subpopulation was performed independently using the standard algorithm with the T helper subset as an example. Using the fluorescence intensity of CD44 and CD62L, the relative number (%) of central (CD44+CD62L+) and effector (CD44+CD62L-) memory cells (gate Tcm and Tem, respectively) was determined.
